# Pilot longitudinal observational study of the effect of common stress events on the shedding of antimicrobial resistant *E. coli* during the pig and poultry production cycle

**DOI:** 10.3389/fvets.2026.1941023

**Published:** 2026-09-16

**Authors:** Megan E. Rawlins, Francesca Martelli, Claire Oastler, Silvia Stronati, Richard P. Smith

**Affiliations:** 1 Department of Bacteriology, Animal and Plant Health Agency, Addlestone, United Kingdom; 2 Department of Epidemiological Sciences, Animal and Plant Health Agency, Addlestone, United Kingdom

**Keywords:** antimicrobial resistance (AMR), *Escherichia coli*, pig production, poultry production, stress events

## Abstract

This longitudinal observational study sampled six farms (three broiler and three pig finishing farms) before and after selected routine management events with the aim of exploring the impact of stress on antimicrobial resistant *E. coli* shedding. A Poisson generalised linear mixed-modelling (GLMM) approach was used for each species to evaluate differences between shedding of *E. coli* following the events compared to the baseline, and impact of explanatory variables. Whilst shedding varied at the sampling time points, no evidence of significant difference was detected. Our results demonstrate no significant difference in the impact of stress on *E. coli* (for all *E. coli* and resistant *E. coli*) shedding at different time points, contrasting with trends seen in other bacteria. However, due to this being a pilot study, we only included a small number of farms and expansion to include additional farms would be beneficial. Certain temporal and farm-level factors influencing shedding were identified which warrant further investigation.

## 1 Introduction

Antimicrobial resistance (AMR) poses a significant public health and animal health and welfare threat. In the United Kingdom (UK), substantial progress has been made in reducing antibiotic use in livestock production (1). However, while this helps control the development of resistance on farm, AMR bacteria can also enter livestock premises through different environmental sources, and shedding can increase during common production management cycle events, such as thinning, transport to abattoir, and depopulation, which are known to exert stress on livestock (2, 3).

In 2024, the pig sector remained the highest user of antibiotics in the UK compared to other livestock sectors (50 mg/kg), and broilers had a usage of 4.7 mg/kg (4). AMR continues to be a concern in both sectors, with fully susceptible *E. coli* isolates from broilers decreasing from 45% in 2022 to 32% in 2024, and *E. coli* with limited treatment options identified in 22% of pig isolates in 2024 (4).

Potential increase of bacterial shedding post-stressful events is due to multiple factors, with mechanisms including immunomodulation playing a role (5). Animal level factors such as age and concurrent diseases can also influence bacterial shedding.

This novel observational pilot study aimed to explore the impact of stress on AMR *E. coli* shedding from poultry (broilers) and pigs (finishers) after stressful events in their production cycles. Improving knowledge in this area would support formulation of evidence-led advice to pig and poultry producers and enable a better understanding as to the potential human health implications via the food chain.

## 2 Method

Broiler and pig finishing production types were chosen as they undergo several events which can cause stress within a short period of time, meaning that stressful events could be assessed during the life of a production cycle and making them easy to sample longitudinally. They also represent a large proportion of the production types in the pig and poultry sector in the UK and are commonly consumed products of animal origin which pose a potential food-borne zoonotic disease transmission risk.

Six farms in total were included in the study: three broiler (Farms 1–3) and three pig farms (Farms 4–6). Several approaches were used to voluntarily recruit farms. Pig finishing farms were recruited via contact (either via letter, email or telephone) to farms registered in APHA databases that were suitably located for sampling (within 3 h of the testing laboratory). A total of 118 pig finishing farms were contacted. Broiler farms were recruited via poultry industry groups who distributed information about the study to their members. The three broiler farms were managed by the same integrated company; however, they were epidemiologically distinct units.

Samples were collected from the six study farms at different timepoints: at least one week before the first stress event (baseline); post-thinning (poultry only); post-depopulation (after the pigs/poultry had been loaded and sent to abattoir); and post-transport to abattoir (the lorry which had transported them to the abattoir was sampled). At baseline sampling, broilers were aged between 20 and 30 days old, and pigs between 18 to 30 weeks of age. Samples were collected between September 2022–February 2024, with each farm’s start depending on farm production schedules and farmer availability. Post-transport to the abattoir samples were collected for Farm 6, however, due to logistical delays the samples were unrefrigerated for 48 h and were not processed within the required time frame, with a potential impact on *E. coli* counts. Results from this timepoint for Farm 6 were therefore excluded from the final analysis. Information on pre-slaughter feed withdrawal practices for broilers was not systematically collected and was therefore not included as a variable in the statistical analysis. The pig farms reported no feed withdrawal prior to depopulation, so this variable was also not considered in our analysis.

Pooled faecal samples were collected from the same broiler house or batch of finisher pigs at each sampling visit. A sampling size of six pooled faecal samples were collected at each visit, to achieve a 95% confidence interval (CI) and a detection limit of 5% *E. coli* spp. (6). These were collected from the floor of the animal accommodation housing the group/flock to be sampled. The floor of the housing was split evenly into six quadrants, and two pinches of faeces taken from five evenly distributed locations in the quadrants. For post-transport sampling, the same approach was applied to the floor of the transport lorry for pigs, and for poultry, 60 individual faecal samples were taken at evenly distributed points from the lorry and transport crates and pooled into six samples in the laboratory.

Samples were processed on the same day of receipt. Pooled faeces were suspended in phosphate-buffered saline (PBS) solution and 10-fold dilutions to −4 prepared (neat, −1, −2, −3 and −4). 100ul of each dilution was spread plated onto three selective agar plates; CHROMagar ECC (without antibiotic) and two CHROMagar ECC plates with antibiotics added. Plates were incubated for 24 ± 3 h at 37 ± 1 °C and colony counts to determine log colony-forming units per gram (cfu/g) of faeces were undertaken following incubation. Suspect *E. coli* colonies were confirmed by sub-culture of a single colony onto a MacConkey plate and incubating plates for 24 ± 3 h at 37 ± 1 °C. Following incubation on the MacConkey plate, a selected colony was sub-cultured onto a 5% sheep blood agar (SBA) plate for purification. Indole and oxidase tests were performed for biochemical confirmation of *E. coli*. A single colony was then frozen down on bead stocks and stored at −80 °C.

Selection of antibiotics for addition to the CHROMagar ECC plates was achieved by screening the baseline samples to identify which antibiotics *E. coli* isolates demonstrated the most resistance to. Antibiotics in the screening panel were chosen either due to their veterinary importance (ampicillin, nalidixic acid, gentamicin, tetracycline, sulfamethoxazole, apramycin) or importance to human health [ciprofloxacin and cefotaxime; both of which are critically important antibiotics (7)]. The panel of antibiotics used for screening poultry farms was 8 mg/L ampicillin, 32 mg/L nalidixic acid, 1 mg/L cefotaxime, 0.5 mg/L ciprofloxacin and 2 mg/L gentamicin. The panel of antibiotics used for screening pig farms was 8 mg/L ampicillin, 8 mg/L tetracycline, 32 mg/L sulfamethoxazole (Farm 4 only), 0.5 mg/L ciprofloxacin (Farms 5 and 6 only), 32 mg/L apramycin (Farms 5 and 6 only) and 1 mg/L cefotaxime (Farms 5 and 6). For Farm 4, antibiotic selection was based on historic antimicrobial resistance data available for this farm from previous research studies (this was not a factor in deciding antimicrobials for the other farms as they had not historically participated in our research studies). The concentration of antibiotics used in selective agar plates was based on EUCAST clinical breakpoints for Enterobacterales, where data was available (8). For Tetracycline, *E. coli* EUCAST COFF values were used; for Apramycin the breakpoint proposed by the Danish Integrated Antimicrobial Resistance Monitoring and Research Programme (DANMAP) was used (9); and for Sulfamethoxazole data from The European Union Summary Report on Antimicrobial Resistance in zoonotic and indicator bacteria from humans, animals and food in 2018/2019 was used (10).

## 3 Results

On screening samples, *E. coli* resistant to ampicillin and nalidixic acid were detected from the poultry farm samples, as well as *E. coli* resistant to ciprofloxacin, gentamicin, or cefotaxime. From the pig farms, *E. coli* resistant to ampicillin and tetracycline were detected, along with *E. coli* resistant to sulfamethoxazole (Farm 4 only), ciprofloxacin (Farm 5 only) or apramycin (Farms 5 and 6 only). Based on these baseline sample results, faeces from poultry farms were plated onto CHROMagar ECC agar containing either 8 mg/L ampicillin, or 32 mg/L nalidixic acid or CHROMagar ECC agar (no added antibiotics) only. Faeces from pig farms were plated onto CHROMagar ECC agar containing either 8 mg/L ampicillin, 8 mg/L tetracycline, or CHROMagar ECC agar (no added antibiotics) only.

The mean log cfu/g for the three broiler and finishing farms were variable across the different time points, with no trend observed (Figure 1). There was a greater variation in mean log cfu/g in poultry farms, ranging from 4.0–8.1 log cfu/g, compared to pig farms (4.1–6.4 log cfu/g) from all samples collected at the different time points for pigs and poultry. The highest value for broiler farms was 8.1 log cfu/g for all *E. coli*, and for ampicillin resistant *E. coli*, at the baseline sampling timepoint (Farm 2). For pig finishing farms, the highest value was 6.4 log cfu/g for all *E. coli* at the post-depopulation timepoint (Farm 6). The percentage of resistant *E. coli* shed as a total of all *E. coli* isolates for each of the timepoints was calculated (Figure 2). For all poultry farms, ampicillin resistant *E. coli* were more prevalent across all sampling timepoints when compared to nalidixic acid resistant *E. coli*. There was a large reduction in nalidixic acid resistant *E. coli* in broiler Farm 3 post-depopulation compared to post-thinning (24.3% decrease), but this subsequently increased by 17.5% at the next sampling timepoint (post-transport). For two of the finishing pig farms (Farms 4 and 5), tetracycline resistant *E. coli* were more prevalent across all timepoints compared to ampicillin resistant *E. coli*. In comparison, for Farm 6, ampicillin resistant *E. coli* were the most prevalent.

**Figure 1:**
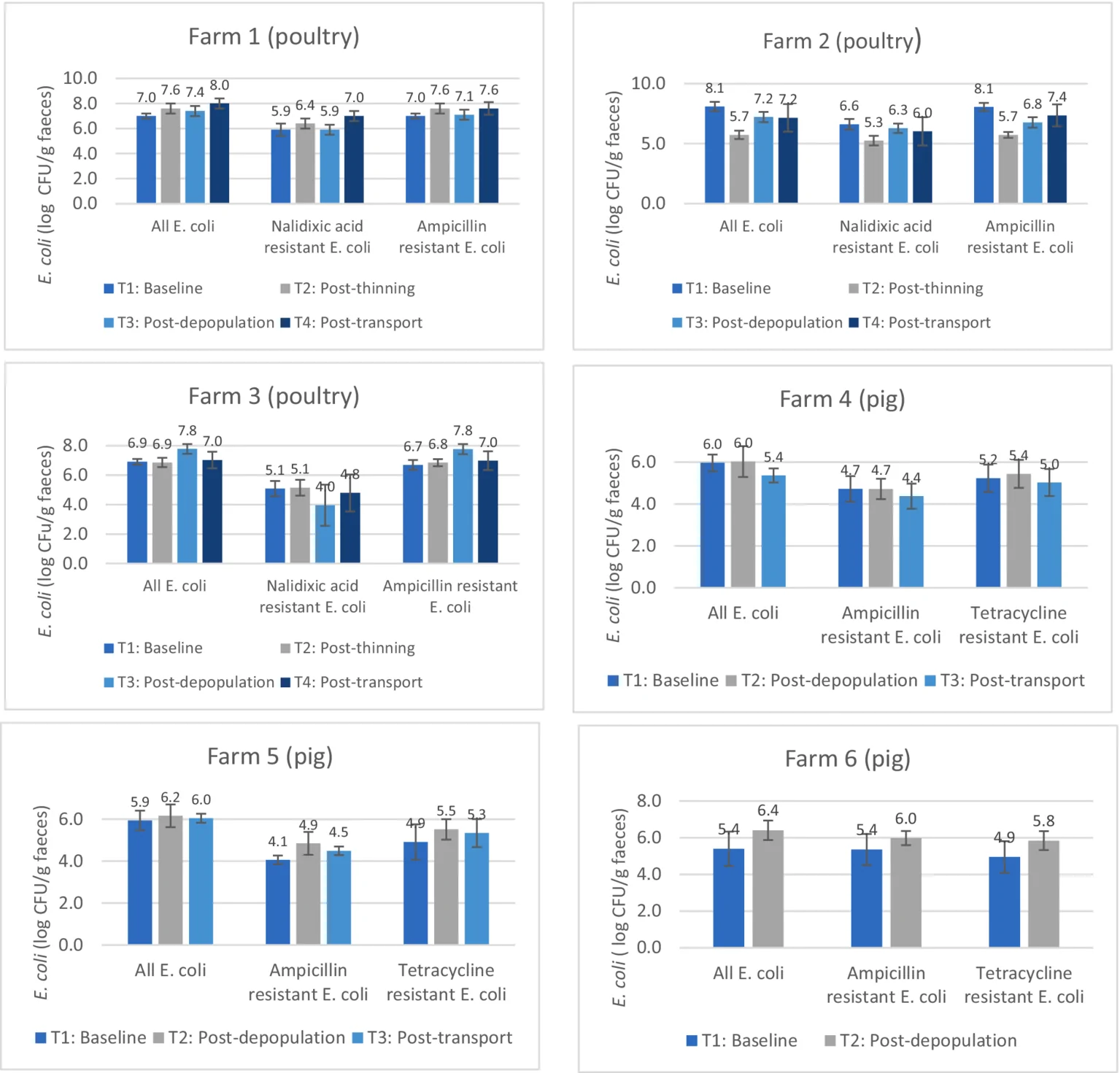
Mean colony-forming unit per gram of faeces (cfu/g) and standard deviation (error bars) for the six study farms (three broiler and three pig farms) at sampling time-points.

**Figure 2:**
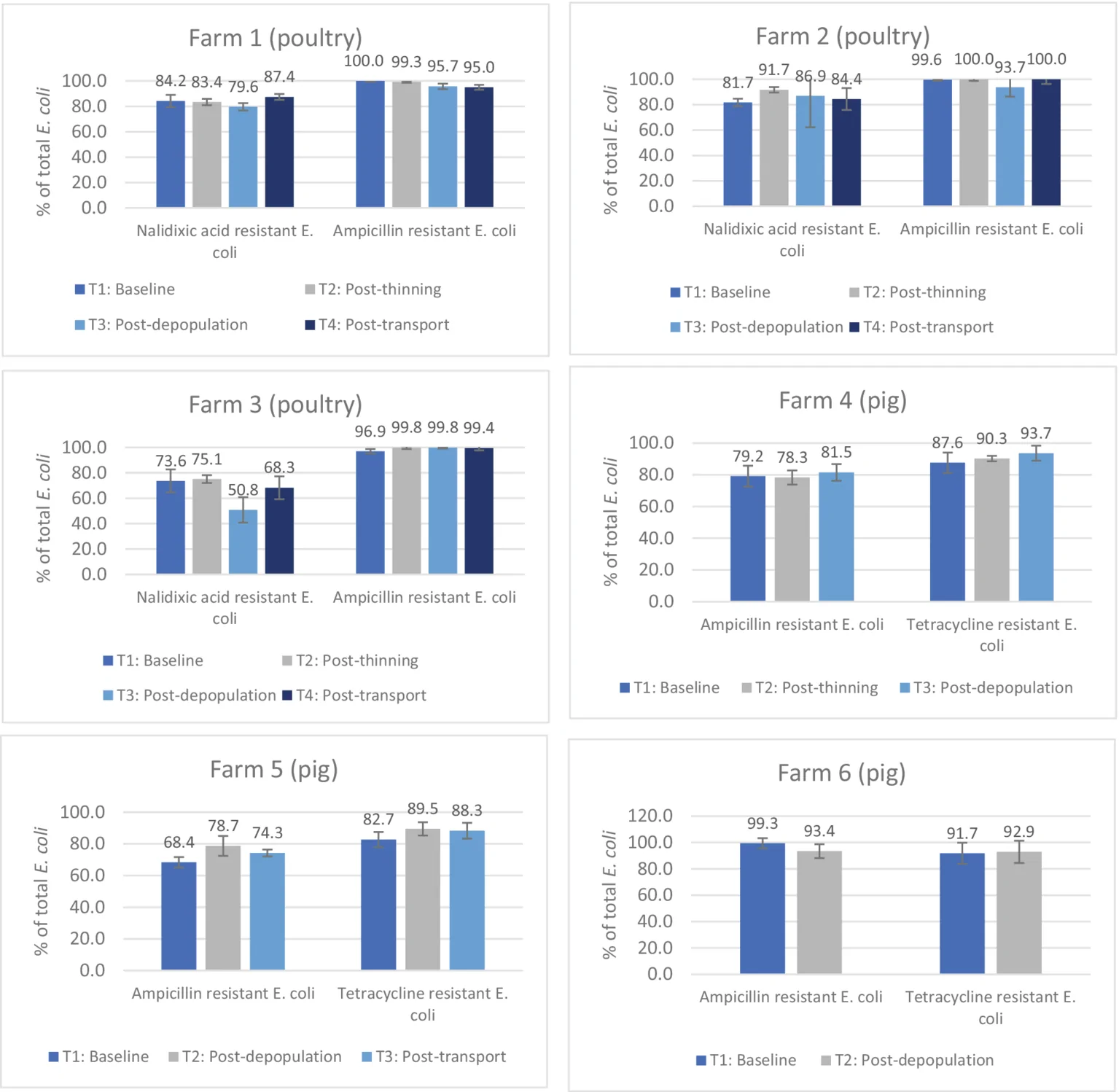
Prevalence (%) of *E. coli* resistant to antibiotics tested, as a proportion of all *E. coli* present in the samples for the six study farms (three broiler and three pig farms) at sampling time-points.

A Poisson generalised linear mixed-modelling (GLMM) approach was used to evaluate differences between shedding of *E. coli* (for all *E. coli* and resistant *E. coli*) at the different stress events compared to the baseline. Separate models were created for pig and poultry farms at the sample level, with farm added as a random effect to account for inter-farm variability. Separate models were fit for all *E.* coli, ampicillin resistant *E. coli* and nalidixic or tetracycline resistant *E.* coli for pig and poultry farms. To assess potential overdispersion in the Poisson GLMM models, we fitted a negative binomial mixed-effects model with the same fixed and random effect structure and evaluated the dispersion parameters. The estimated dispersion parameters were consistently large, indicating negligible overdispersion so we retained the Poisson GLMM models. A random intercept for sampling visit, nested within farm, was considered. However, this was not pursued as the farm-level effect in most GLMM models showed negligible variance, indicating limited clustering between farms. Additionally, due to the limited sample size and small number of observations per sampling visit this could not be reliably estimated. We interpreted the incidence rate ratios (IRRs) to reflect the multiplicative change in expected colony counts.

There was no significant difference in *E. coli* shedding counts at the different timepoints compared to the baseline for both pig and poultry farms. For pig farms, estimated incidence rate ratio (IRR) exceeded 1 for each timepoint, although 95% CI included less than 1 for all.

Flock size (poultry only), time (days) since baseline sampling, month sampled, and season sampled were included as explanatory variables. On univariate analysis for the broiler data (excluding timepoint), sampling in May (IRR: 0.78 (95% CI: 0.59–0.97), *p* = 0.013) and Spring (IRR: 0.75 (95% CI: 0.56–0.94), *p* = 0.003) were significant variables (lower expected colony count than compared to baseline). Additionally, flock size of 45,500 (IRR: 1.27 (95% CI: 1.08–1.46), *p* = 0.012) or 95,500 (IRR: 1.33 (95% CI: 1.14–1.52), *p* = 0.003) birds were significant variables (higher expected colony count than compared to baseline). However, these were significant for nalidixic resistant *E. coli* shedding only. None of the variables considered were significant for pigs on univariate analysis. Inclusion of explanatory variables into the GLMM with timepoint did not result in interaction with or alter the IRR at the different timepoints compared to the baseline for either pig or poultry farms. The significant explanatory variables from the univariate analysis remained significant when incorporated into the GLMM with timepoint.

## 4 Discussion

This field study demonstrated that whilst there was variation in shedding at the sampling timepoints, there was no significant difference between *E. coli* shedding post-stressful events (for all *E. coli* and resistant *E. coli*) compared to baseline, suggesting that *E. coli* shedding in pigs and poultry was not affected by the stressful events investigated in this study. Whilst there was some variability in the proportion of *E. coli* isolates which were resistant to the antibiotics tested at the different timepoints, the overlapping of CI suggested no significant difference in the proportion of resistant *E. coli* shed.

Observed higher levels of *E. coli* shedding in poultry compared to pigs agrees with a previous study (although statistical analysis to see if a significant difference existed was not undertaken for this study due to the limited scale of the study population) (11). The same study arbitrarily classified > 4.00 log cfu/g as high-density shedders, meaning that at all timepoints in this study, farms sampled fell into this category. Hygiene measures at the abattoir will therefore be critical to mitigate risks of food chain contamination. Further research exploring the levels of *E. coli* shedding in pig and poultry units on a broader scale would help with producing evidence-based categories for shedding levels.

Previous studies have demonstrated increasing *E. coli* counts in pigs particularly post-handling events (12) and reference stress as a potential factor increasing *E. coli* shedding in broilers. Prior research has predominantly focussed on exploring the effects of a range of stressors on *E. coli* shedding, rather than elucidating this specific effect on resistant *E. coli*, as was the objective of our study (3, 13). The impact of stress on shedding of bacterium from livestock has been demonstrated with other pathogens including Salmonella spp., and it was anticipated that a similar trend would be observed in our study. However, the lack of detection of this trend may reflect the limitations of this pilot study, discussed below in further detail, rather than there truly being no impact.

Whilst the time point of sampling was not statistically significant for AMR *E. coli* shedding, temporal and farm-level factors including month sampled and flock size were significantly associated with shedding of some resistant *E. coli* in broiler farms, suggesting that dynamics of shedding for different AMR *E. coli* may vary. Sampling in May and Spring significantly reduced *E. coli* shedding, whereas a larger flock size significantly increased shedding (although the increase was marginal). However, these variables were likely significant and show the observed trends because Farm 3 (broiler) which had the smallest flock size (36,500 birds) and was the only farm sampled in May and Spring and had a lower mean shedding of nalidixic resistant *E. coli* across all time-points when compared to the other broiler farms. There was a large reduction in nalidixic acid resistant *E. coli* in Farm 3 post-depopulation, but this had increased post-transport. This is interesting given the short time between depopulation and transport (sampled same day) and could reflect the intermittent nature of *E. coli* shedding even under stressful conditions. Resistance was detected to critically important antibiotics (ciprofloxacin and/or cefotaxime) in some of the pig and broiler farms in this study, which is a potential public health risk. Differences in the proportion of resistant isolates between broiler and pigs and the different study farms reflects the environmental variability and potentially different antimicrobial usage practices (although pre-sampling antimicrobial usage data was not collected, so it is not possible to link usage to resistant *E. coli* isolates). Inclusion of additional farms would enable further exploration of the variability we observed and further explore any potential impacts. Following subsequent flocks/herds at the same farms would enable inter-farm comparability to reduce the impact of extraneous variables.

Feed withdrawal has been demonstrated to increase bacterial shedding in cattle. Pig farms in this study did not practice feed withdrawal (8–12 h) before depopulation so we did not explore this stress impact. It is commonplace for broilers to have feed withdrawn for up to 12 h prior to slaughter, although we did not gather information in this study of feed withdrawal times in the flocks studied. Feed withdrawal should therefore be considered as a potential explanatory variable for *E. coli* shedding, which could not be assessed in this study.

This study was limited by the small number of farms used and resultant low statistical power due to this. Although all farms were expected to be representative of commercial UK production, the poultry farms came from the same integrated company, and it is unknown how representative the farms were. Therefore, whilst some factors were significant and there were some trends for *E. coli* shedding observed, it is challenging to draw any firm conclusions. Gathering more systematic on-farm data would enable additional explanatory variables for *E. coli* shedding to be explored in more depth. A further limitation of the study is the assumption that the ‘baseline’ state reflects a low stress state, when in practice other variables could be exerting stress.

Initial findings are potentially positive from a public health perspective, in that there appears to be no large increase in *E. coli* and AMR *E. coli* shedding during pre-slaughter stressful events, which could pose a risk via contamination during food chain processing. However, these findings are preliminary, and our study has low statistical power. Levels of *E. coli* shedding in this study were high, although further exploration is needed to quantify the risks this may pose. This study presents some initial results, which could assist with developing future studies on this topic.

## Data Availability

The datasets presented in this article are not readily available because as a UK government agency, the authors need to assess each request for the data set. Requests to access the datasets should be directed to megan.rawlins@apha.gov.uk.

## References

[1] El Nagar A Dewé TCM Broadfoot F Teale C Smith RP. Decreasing antimicrobial resistance in representative UK livestock species was associated with reduced total sales of antimicrobials in the last decade. J Antimicrob Chemother. (2025) 80:1902–6. doi: 10.1093/jac/dkaf145, 40471099

[2] Koutsoumanis K Allende A Álvarez-Ordóñez A Bolton D Bover-Cid S Chemaly M . Role played by the environment in the emergence and spread of antimicrobial resistance (AMR) through the food chain. EFSA J. (2021) 19:e06651. doi: 10.2903/j.efsa.2021.6651 PMC8210462 34178158

[3] Rostagno MH. Can stress in farm animals increase food safety risk? Foodborne Pathog Dis. (2009) 6:767–76. doi: 10.1089/fpd.2009.0315, 19737056

[4] VMD UK Veterinary Antibiotic Resistance and Sales Surveillance Report. New Haw, Addlestone: Veterinary Medicines Directorate. (2025)

[5] Jones PH Roe JM Miller BG. Effects of stressors on immune parameters and on the faecal shedding of enterotoxigenic *Escherichia coli* in piglets following experimental inoculation. Res Vet Sci. (2001) 70:9–17. doi: 10.1053/rvsc.2000.0436, 11170846

[6] EFSA (European Food Safety Authority). Analysis of the Baseline Survey on the Prevalence of Campylobacter in Broiler Batches and of Campylobacter and Salmonella on Broiler Carcasses in the EU, Part A: Campylobacter and Salmonella Prevalence Estimates. EFSA J. (2010) 8: 1503. doi: 10.2903/j.efsa.2010.1503 PMC13090778 42006083

[7] WHO. WHO’s List of Medically Important Antimicrobials: a risk management tool for mitigating antimicrobial resistance due to non-human use. Geneva: World Health Organization. (2024)

[8] EUCAST The European Committee on Antimicrobial Susceptibility Testing. Breakpoint Tables for Interpretation of MICs and Zone Diameters. Version 14.0, 2024. (2024). Available online at: http://www.eucast.org (Accessed September 08, 2026).

[9] Jensen VF Jakobsen L Emborg H d Seyfarth AM Hammerum AM. Correlation between Apramycin and Gentamicin use in Pigs and an Increasing Reservoir of Gentamicin-Resistant Escherichia coli. J Antimicrob Chemother. (2006) 101–7. doi: 10.1093/jac/dkl201 16709594

[10] European Food Safety Authority, European Centre for Disease Prevention and Control. The European Union one health 2020 zoonoses report. EFSA J. (2021) 19:e06971. doi: 10.2903/j.efsa.2021.6971, 36329690 PMC9624447

[11] Horton RA Randall LP Snary EL Cockrem H Lotz S Wearing H . Fecal carriage and shedding density of CTX-M extended-Spectrum β-Lactamase-producing *Escherichia coli* in cattle, chickens, and pigs: implications for environmental contamination and food production. Appl Environ Microbiol. (2011) 77:3715–9. doi: 10.1128/AEM.02831-10 PMC3127594 21478314

[12] Dowd SE Callaway TR Morrow-Tesch J. Handling may cause increased shedding of *Escherichia coli* and total coliforms in pigs. Foodborne Pathog Dis. (2007) 4:99–102. doi: 10.1089/fpd.2006.53, 17378714

[13] dos Santos VM Oliveira GD Salgado CB Pires PG Santos PH McManus C. Outcomes of microbiological challenges in poultry transport: a mini review of the reasons for effective bacterial control. Microbiol Res. (2024) 15:962–71. doi: 10.3390/microbiolres15020063

